# A dual layer broadband radar absorber to minimize electromagnetic interference in radomes

**DOI:** 10.1038/s41598-017-18859-w

**Published:** 2018-01-10

**Authors:** Thtreswar Beeharry, Riad Yahiaoui, Kamardine Selemani, Habiba Hafdallah Ouslimani

**Affiliations:** 1 Laboratoire Energétique Mécanique Electromagnétisme, Université Paris Nanterre, Ville d’Avray, 92410 France; 2 Constructions Mécanique de Normandie, Systems department, Cherbourg, 50105 France

## Abstract

A thin broadband dual-layer radar absorber based on periodic Frequency Selective Surfaces (FSS) to tackle Electromagnetic Interference (EMI) in radomes is presented in this article. The proposed structure consists of periodically arranged metallic patterns printed on two dielectric substrates separated by an optimized air gap. Under normal incidence, the proposed structure exhibits at least 89.7% of absorption in the whole band of 4.8 GHz to 11.1 GHz for both Transverse Electric (TE) and Magnetic (TM) polarizations. For oblique incidences, a very slight decrease in the bandwidth is observed in the upper frequency band until 30° and the absorption remains very interesting for higher incidences. The structure is *λ*/7.2 (*λ* is the wavelength in free space) thin compared to the center frequency (8.2 GHz). In addition, parametric studies have demonstrated that at least 90% of absorption can be produced with our structure by adjusting the thicknesses of the dielectric substrates. Another issue that is presented and discussed in this paper is a new approach for evaluating the performance of absorbers. In fact, studies show that the absorber can compete with other recent broadband absorbers. After fabricating the structure, the measurements were found to be in good agreement with the simulation results.

## Introduction

Electromagnetic wave absorbers cover a range of applications in defense systems^1^ such as reduction of radar cross section, stealth applications, reduction of EMI between electronic equipments and others. On ships for example, several antennas and radars are mounted inside radomes which may contain metallic objects such as cable ducts. Reflected electromagnetic waves due to these metallic objects cause EMI critical issues. The metallic ducts also cause indirect echoes, shadow and blind zones. Covering metallic parts with radar absorbers can decrease considerably the reflections, and hence the indirect echoes. For practical reasons, broadband thin absorbers covering part or the whole frequency band of radars and antennas are best suitable for these applications. Jaumann absorbers^2–4^ and Salisbury screens^5–7^ are very good examples of radar absorbers. Multilayer Jaumann absorbers suffer from large thickness and bulkiness as each layer produces a single narrow band resonance. Based on metamaterial technique^8–13^, FSS are well tailored periodically arranged resonant structures printed on dielectric substrates^14–17^. FSS improve the bandwidth, reduce bulkiness and thickness of the absorber compared to Jaumann absorbers. FSS based absorbers suffer from a narrow bandwidth (high quality factor; *Q* = *f*_0_/Δ*f*_−3*dB*_) because of their resonant structures. In order to enlarge the bandwidth, two of the popular techniques used, consist of incorporating resonating elements working at nearby frequencies by arranging then on the same plane^18–21^ or by using multi layers^22–24^. Both techniques lead to a drop in the absorption (sometimes the reflection, *S*_11_ > −10 dB) at certain frequencies in the bandwidth. Using several elements on the same plane is very difficult to implement due to lack of space in a small unit cell. Moreover, using different resonating elements on a single unit cell can lead to an asymmetrical design and hence degrading the performance considerably for oblique incidences and different polarizations. By following the same reasoning a multilayer structure can considerably increase the total height of the absorber. Using well customized magnetic materials^25^, can decrease the thickness^26^ but they can be very expensive. For these reasons, designing thin broadband radar absorbers with easily available dielectrics especially for low frequencies (VHF/UHF) and the gigahertz regime (radio frequency and microwave) is very complicated and is a challenging topic. In this paper, we present a thin ultra-broadband radar absorber operating in the band of 4.8 GHz–11.1 GHz achieving more than 89.7% of absorption in the whole band for both TE and TM polarizations at normal incidence. We also demonstrate that by decreasing the total thickness of the absorber, more than 90% of absorption is obtained without affecting consequently the frequency band. The absorption remains unchanged until 30° showing an absorption efficiency of more than 89.7% with a slight decrease in bandwidth (reduction of 1.1 GHz in the upper band). Results remain very interesting until 50° of incidence. The designed structure achieves a low profile which can reach thickness value that is very close to the theoretical limit^26^ as detailed later in the paper.

## Results

### Design and simulation results

**Figure 1:**
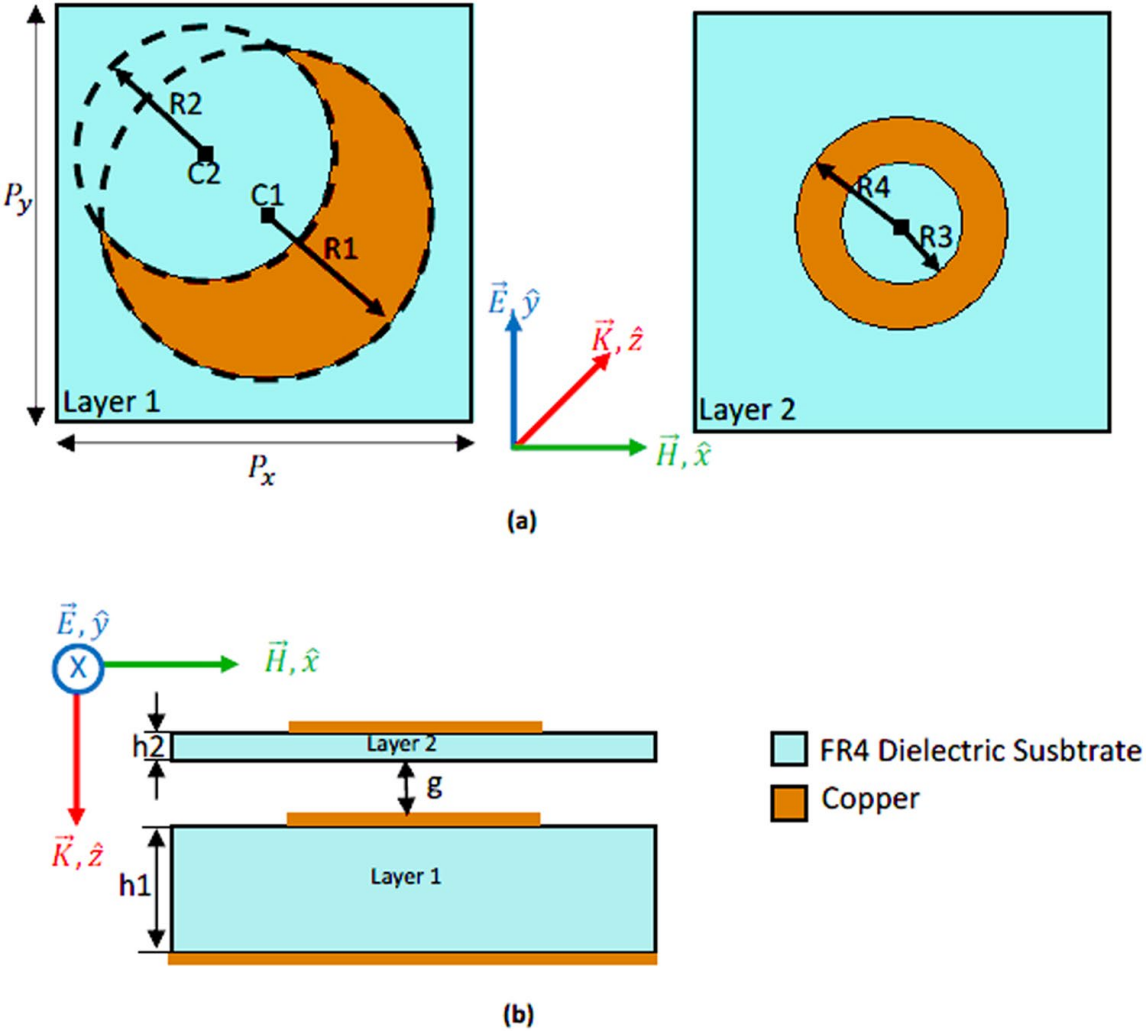
(**a**) On the Left hand side: Top view of layer 1 with the HMSR. On the right hand side: Top view of layer 2 with the RSR. (**b**) Perspective view of the proposed absorber. The first layer is backed with a metallic ground. The second layer is separated from the first layer by an air gap of 1.5 mm.

The unit cell of the proposed dual layer absorber is depicted in Fig. 1. The absorber is composed of two layers separated with an optimized air gap of thickness, *g* = 1.5 mm. For the simulations, copper having $5.8\ast {10}^{7}\,{\rm{S}}/{\rm{m}}$ of electric conductivity and 0.0175 mm of thickness is used for the metallic patterns and ground plane, and dielectric FR4 of relative permittivity *ε* = 4.2 and loss tangent *δ* = 0.018 is used for the two dielectric substrates. The first FR4 layer, whose thickness, *h*1, is equal to 3.2 mm, is backed with a ground plane to prevent transmission. On top of the first layer, a half-moon-shaped resonator (HMSR) is deposited. The HMSR is obtained by removing, from a circular patch of radius *R*1 = 5.45 mm and center C1 (*x* = 0 mm, *y* = 0 mm), a circular section having radius *R*2 = 4.2 mm and center, C2 (*x* = 2 mm, *y* = −2 mm). A ring-shaped resonator (RSR), of outer radius *R*4 = 3.5 mm, and inner radius *R*3 = 2 mm, is deposited on top of the second FR4 layer which has a thickness, *h*2, equal to 0.5 mm. The unit cells (HMSR and RSR) are arranged in periods of *P*_*x*_ = *P*_*y*_ = 13.75 mm.

**Figure 2:**
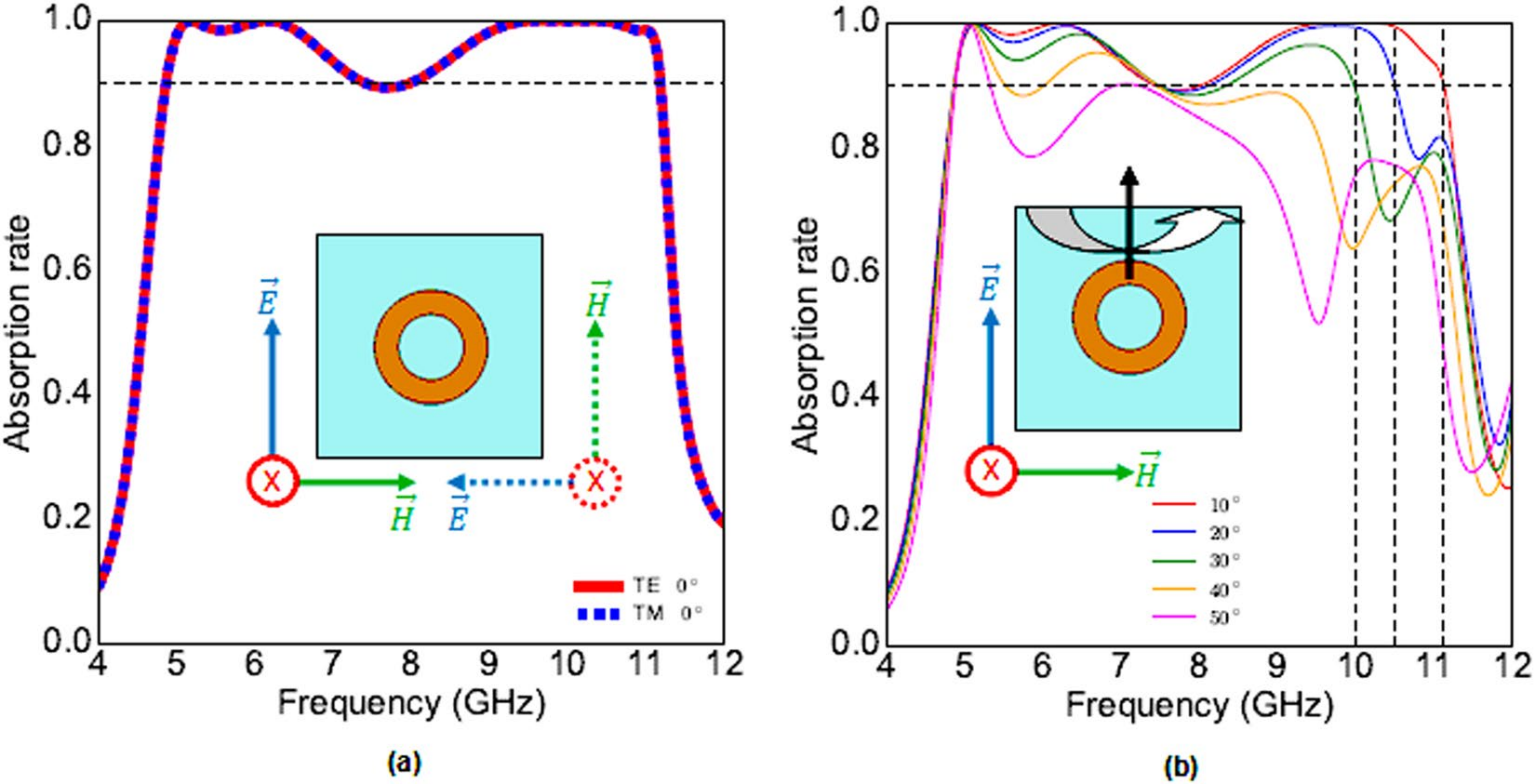
(**a**) The absorption ratio of TE and TM for normal incidence. (**b**) Absorption ratio of TE mode for oblique incidences.

Absorption is given by the formula *A*(*ω*) = 1 − |*S*_11_(*ω*)|^2^ − |*S*_21_(*ω*)|^2^. The first layer of our structure is backed by a metallic plate so no transmission occurs. Hence, in our case, *A*(*ω*) = 1 − |*S*_11_(*ω*)|^2^. The absorption of the whole structure is presented in Fig. 2a. As shown in Fig. 2a, for normal incidence, the absorption rate is more than 89.7% in the whole band of 4.8 GHz to 11.1 GHz for TE and TM modes. Simulation results for oblique incidences of linearly polarized TE wave are shown in Fig. 2b. For oblique incidences until *θ* = 30°, the absorption remains more than 89.7% but the bandwidth is very slightly decreased. For 40° of incidence, the absorption rate is higher than 80% in the band of 4.8 GHz–9.5 GHz and remains above 64% until 11.1 GHz. For 50° of incidence, the absorption is deteriorated but remains significant (>78%) in the band of 4.8 GHz–8.7 GHz and more than 50% in the band of 8.7 GHz–11.1 GHz. These results remain extremely interesting for a large band absorber. The results for oblique incidences concerning TM polarization (not shown) are the same as those of TE polarization.

### Absorption mechanism

**Figure 3:**
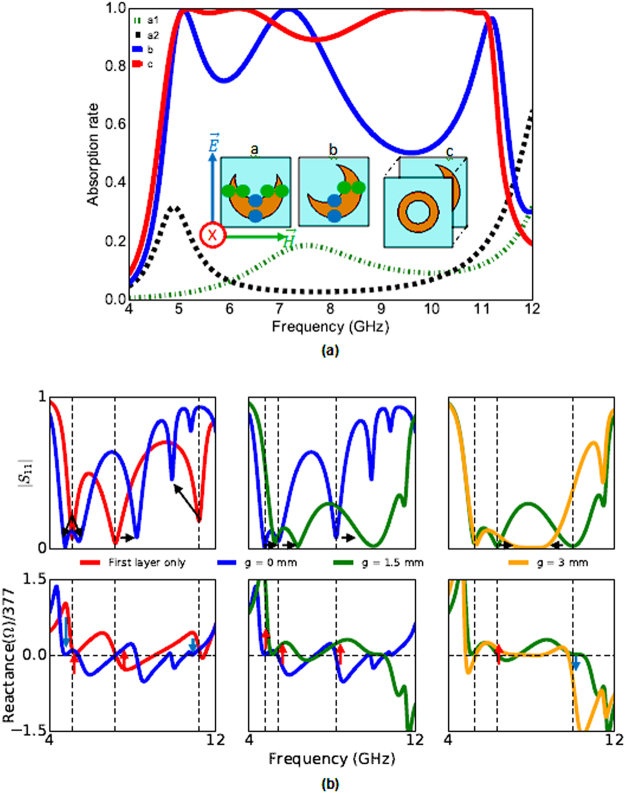
(**a**) Absorption of each layer. Dotted green and black curves represent the TE and TM modes of layer 1 respectively as oriented in case ‘a’. The continuous blue curve shows the TE mode of layer 1 as oriented in case ‘b’. For cases ‘b’ and ‘c’, TE and TM modes are equal. (**b**) Are shown: In the first row, the reflexions (*S*_11_) of different values of g. In the second row, the reactances of different values of g.

To understand the absorption mechanism of our structure, we will first study the behavior of each layer. Figure 3a depicts the absorption of each layer and the inset shows three different cases. In cases ‘a’ and ‘b’ only the first layer backed with a metallic plate is studied. In case ‘c’ the whole structure the (metallic ground, first layer, air gap and second layer) is simulated. In the three cases absorption is calculated by *A*(*ω*) = 1 − |*S*_11_(*ω*)|^2^. For the first layer, when the HMSR is oriented as in case ‘a’ of Fig. 3a, the segment of the patch excited by the electric field (green points on the patch) is not the same as the segment excited by the magnetic field (blue points on the patch). Hence, the reflection for TE represented by curve ‘a1’ and TM represented by curve ‘a2’ are not the same. Moreover, a very low absorption is observed for both TE and TM modes. Therefore, the patch oriented as in case ‘a’ is not efficient. When the HMSR is rotated by 45°, as in case ‘b’, the structure becomes more interesting as the electrical and magnetic field interact with exactly the same segments of the patch. TE and TM modes become equal as shown by curve ‘b’ (only TE is plotted to simplify the figure).

Furthermore, the absorption is considerably increased. The absorption is at least 70% between 4.7 GHz and 8.2 GHz and at least 50% between 8.2 GHz–11.6 GHz. In order to further reduce the reflection in the band 8.2 GHz–11.6 GHz, the second layer is introduced. When layer 2 is placed at 1.5 mm above the first layer whose patch is rotated by 45°, as shown in case ‘c’, the reflection of the whole band is reduced. As shown by curve ‘c’, the absorption is at least 89.7% for both TE and TM modes between 4.9 GHz and 11.1 GHz. We can also observe a shift in the resonant frequencies when we compare the absorption rates of the first layer and the final structure. In order to understand this phenomenon, a parametric study on the thickness of the air gap, g, has been done. The study included the reflections and the imaginary parts of the effective impedances of the first layer and of different thicknesses of g. Frequency dependent input impedance of the structure is given by the formula *Z*(*ω*) = (1 + *S*_11_(*ω*))/(1 − *S*_11_(*ω*)). The real part and imaginary part of the normalized effective input impedance must be close to 1 and 0 respectively in order to match the impedance of free space (377 Ω or 1 if normalized). The sign of the imaginary part of the effective impedance (reactance) indicates whether the whole structure is having a capacitive or an inductive behavior. If the reactance is positive, the behavior is inductive and if the latter is negative, the structure has a capacitive behavior. A decrease in the reactance value also means that a capacitive coupling have taken place and an increase in the latter means an inductive coupling have taken place. The reflections and the reactances for different values of g under normal incidence of linearly polarized TE wave are plotted in Fig. 3b. The first row of Fig. 3b, corresponds to the reflection and the second row corresponds to the reactance. To begin with, we compare the resonance frequencies of first the layer only (red curve) to that of when the second layer is added and g = 0 mm (blue curve). We can observe that when we add the second layer, the first frequency peak splits into two adjacent resonances, the second resonance shifts to the right and the third resonance shifts tho the left (first column, first row of Fig. 3b). The splitting of the first resonance is due to two adjacent inductive (blue arrows) and capacitive (red arrows) couplings brought by the second layer (first column, second row). When the gap is increased from 0 mm to 1.5 mm (green curve), the inductive coupling increases and all the frequency resonances move towards higher frequencies (second column of Fig. 3b). When the gap is increased from 1.5 mm to 3 mm (orange curve), the first resonance frequency does not change, the second moves to the right, and the third to the left (third column, first row of Fig. 3b). We can observe from their reactance (third column, second row of Fig. 3b) that an inductive coupling takes place at the second resonance and a capacitive coupling takes place at the third resonance. Hence, adding and air gap and the second layer brings and additional capacitive and inductive coupling which modifies the overall effective impedance of the structure and shifts the resonance frequencies.

**Figure 4:**
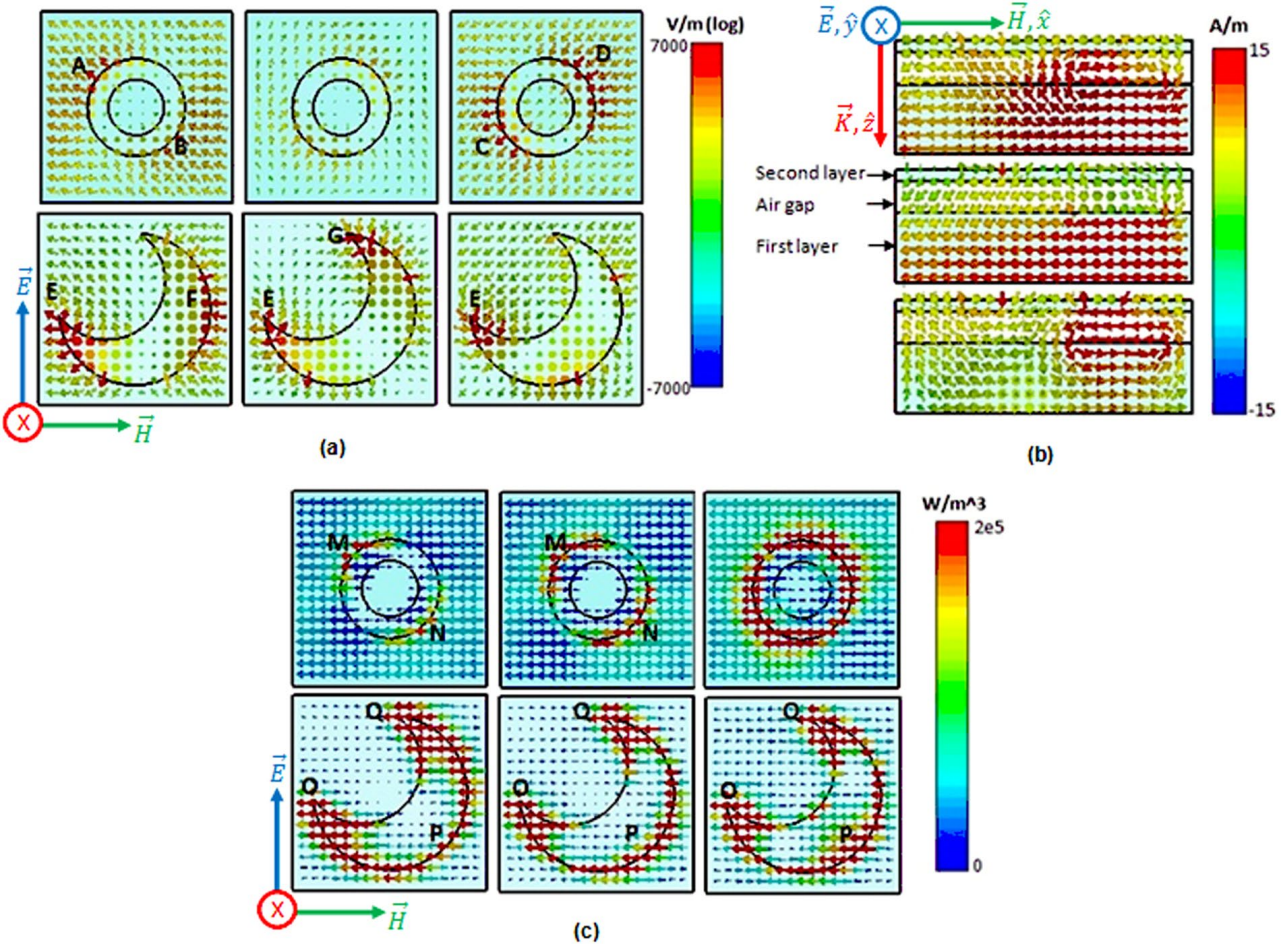
(**a**) Top view of electric field distribution on the first and second layers. (**b**) Perspective view of magnetic field distribution on the first whole structure. (**c**) Top view of power loss density on the first and second layers.

To further understand the absorption, we will in this section study the electric field (Fig. 4a) the magnetic field (Fig. 4b) distributions and the power loss density (Fig. 4c) on the two layers for normal incidence of linearly polarized EM waves. The three columns of Fig. 4 a and c correspond to the three frequencies of maximum absorption peaks (5.17 GHz, 6.16 GHz and 10 GHz). Their two rows illustrate the first layer with the HMSR and the second layer with the RSR. The three rows of (Fig. 4b) depict the magnetic field distribution on the whole structure at the frequencies of maximum absorption peaks.

For the first absorption peak, the electric resonance is produced around regions A and B of the RSR and around regions E and F of the HMSR (first column of Fig. 4a. We can also observe in the first row of Fig. 4b, a strong magnetic resonance on the two layers and the air gap. Power loss will mainly occur where the current circulation (not shown) is strong. As shown in the first column of Fig. 4c, the surface losses are mainly caused around regions M, N, O, P and Q. For the first absorption peak, the electric resonance is produced around regions E and G of the HMSR as shown in the second column of Fig. 4a. We notice from the second row of Fig. 4b, that a strong magnetic resonance is created only between the HMSR and the ground plane. Surface losses are mainly caused around regions M, N, O, P and Q as shown in the second column of Fig. 4c. For the last absorption peak, the electric resonance is produced around regions C and D of the RSR and around region E of the HMSR (third column of Fig. 4a). In the third row of Fig. 4b, we can see a strong magnetic resonance between the HMSR and the RSR due to the circulation of strong anti parallel currents. The surface losses (third column of Fig. 4c) are produced by the whole RSR and regions O, P and Q of the the HMSR. In the three cases overlapping of electrical (Fig. 4a) and magnetic resonances (Fig. 4b) and surfaces losses (Fig. 4c) due to high intensity of current circulation on the HMSR and RSR lead to strong absorption.

### Experimental results

An experimental prototype is fabricated with 16 × 16 unit cells of dimensions 300 × 300 mm using printed circuit board technology. Both layers (Figure a–c) were fabricated with a margin error of ±10% for the thicknesses. The thicknesses of the metallic ground and resonating elements are of 0.018 mm. Washers of thickness 1.5 mm ±10% were used to create the 1.5 mm air gap between the two layers. Eight holes (four at each end and four in the middle of each edge) of 6 mm were made, and bolts/nuts/screws were used to fix the two layers together.

**Figure 5:**
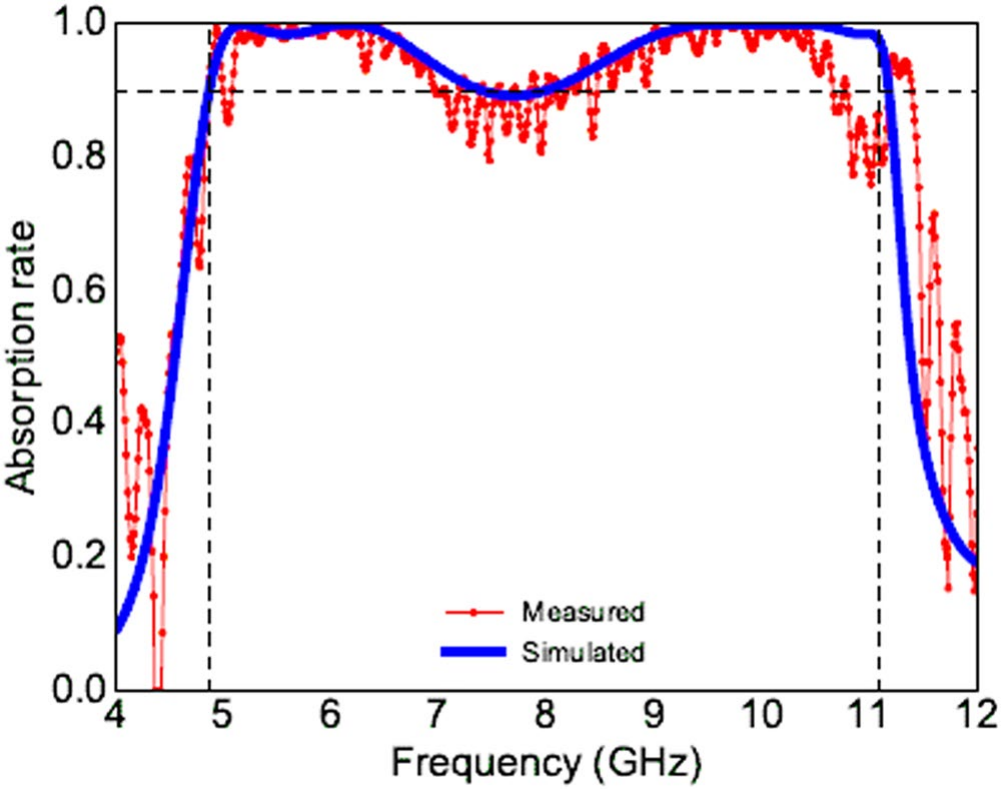
In red, measured absorption. In blue, simulated absorption.

The simulated (blue curve) and the measured (red curve) are plotted in Fig. 5. We can clearly see a very good agreement between both results. Moreover, we can clearly see the three near unity absorption peaks at 5.17 GHz, 6.16 GHz and 10 GHz for the measured absorption. The measured absorption is the same for both TE and TM modes. The thicknesses of the two substrates and the air gap in simulation were chosen such that the fabrication and measurements could be done easily. However, a parametric study is done in the next chapter to illustrate that total thickness of the structure can be reduced and the structure leads more than 90% of absorption band in the range of 4.97 GHz–11.2 GHz.

## Discussion

**Figure 6:**
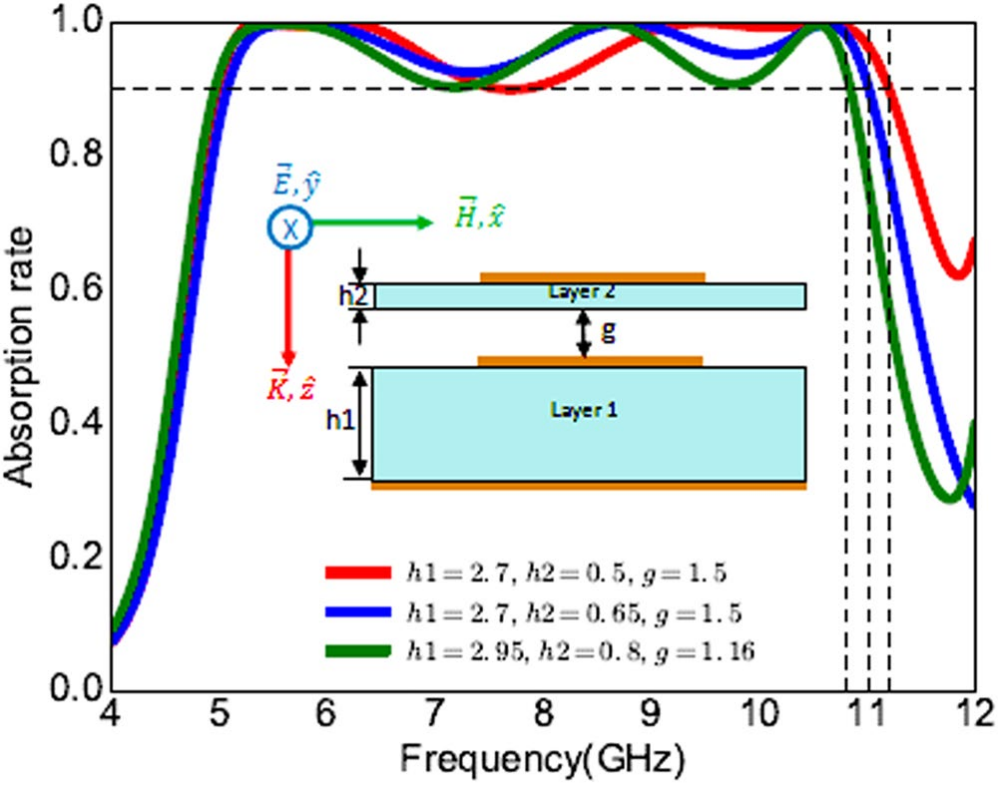
Absorption rate for linearly polarized TE wave of different values of g, h1 and h2. All dimensions are in millimeters.

As discussed earlier, the thicknesses of the two substrates and the air gap in simulation were chosen such that the fabrication and measurements could be done easily and rapidly. Different values of g, h1 and h2 are plotted in Fig. 6. When *h*1 = 2.7 mm, *h*2 = 0.5 mm, *g* = 1.5 mm (red curve), the absorption rate is at least of 90% in the whole band of 4.97 GHz–11.2 GHz. When *h*1 = 2.7 mm, *h*2 = 0.65 mm, *g* = 1.5 mm (blue curve), the absorption is at least of 93% in the whole band of 5 GHz–11 GHz. Finally, when *h*1 = 2.95 mm, *h*2 = 0.8 mm, *g* = 1.16 mm (green curve), the absorption is at least of 90% in the whole band of 4.9 GHz–10.8 GHz. Hence, the different thicknesses of g, h1 and h2 can be selected according the the frequency band and the least amount of absorption required.

The performance of an absorber is often judged upon its −10 dB reflection or 50% absorption rate (full width at half maximum). These two criteria do not take into consideration the thickness of the absorber which can be the most important factor in some applications. A better method to evaluate the performance of an absorber has been proposed by Chang Long^27^. The authors^27^ have used the ratio of −10 dB operational bandwidth to thickness to evaluate the performance of an absorber. It seems that the bigger the ratio is, the better (in terms of performance) the absorber is. We consider this method better than the first two mentioned methods to evaluate the performance of an absorber. However, the problem with this approach is that that we can not compare the performance of two absorbers having exactly the same −10 dB frequency band and having exactly the same thicknesses. Also, with these approaches two absorbers must be compared only if, firstly, they have the same operating frequency range and different thicknesses or, secondly, same thicknesses and different operating frequencies. We often see comparison between absorbers which do not operate in the same frequency range and do not have the same thicknesses. We present a new approach to evaluate the performance of an absorber. According to Rozanov^26^, the minimum theoretical thickness of a non magnetic absorber can be calculated by $d\ge |{\int }_{0}^{\infty }\,ln|\rho (\lambda )|d\lambda |/2{\pi }^{2}$, where *ρ* is the module of the reflexion coefficient, *λ* is the wavelength in free space, and *d* is the theoretical minimum thickness required. This formula takes into account the exact reflexion, and the whole frequency range of the absorber (not the −10 dB frequency range). Our approach to evaluate the performance of a radar absorber consists of doing the ratio of the minimum theoretical thickness that the absorber must have to the real thickness of the structure. The minimum value of this ratio is very close to zero (≈0% in terms of percentage), and its maximum value is one (100% in terms of percentage). The closer to 100% the value is, the closer will be the thickness of the structure to its theoretical thickness. This approach have been used in a few works in which the authors^15^ have used it to have an idea of the minimum thickness value of their absorbers, but, to our knowledge, it has very rarely (not to say never) been exploited to compare absorbers. The advantage of this approach is that we will be able to compare the performances of absorbers which does not operate in the same frequency band as the minimum theoretical thickness to real thickness ratio is a ratio in which the thickness of an absorber is compared to its minimum possible thickness in a first time and then we compare the percentages of other absorbers. The disadvantage of this approach is that the exact S parameters of the absorber must be known and can be time consuming to design and simulate absorbers that we want to compare. In Table 1 we compare the performance of our absorber to some broadband absorbers and we can see that our absorber has the best minimum theoretical thickness to real thickness ratio. We have used the reflexion coefficient for the dimensions that measurements were carried out, that is *h*1 = 3.2 mm, *h*2 = 0.5 mm and *g* = 0.5 mm. In this case we obtain a ratio of 80% which is the best among the compared ratios. As we have shown in the parametric results, h1, h2 and g can be optimized for better absorption. For the case when *h*1 = 2.7 mm, *h*2 = 0.5 mm and *g* = 1.5 mm, the theoretical thickness must be of 4.13 mm in the band of 4 GHz–12 GHz. The theoretical thickness to real thickness ratio is as high as 87.87% in this case.

**Table 1:** Comparison of our absorber to other absorbers.

Broadband Absorbers	Frequency range (GHz)	*t*_*T*_ = Theoretical thickness (mm)	*t*_*R*_ = Real thickness (mm)	(*t*_*T*_/*t*_*R*_) $\ast$ 100
Reference^28^	4–15	1.206	1.54	78.31%
Reference^29^	4–8	1.237	1.6	77.31%
Reference^30^	40–134	0.294	1	29.4%
Reference^27^	6–19	3.43	4.36	78.57%
Reference^31^	5–25	2.55	3.65	69.86%
Reference^32^	6–18	1.64	2.22	73.78%
This work	4–12	4.16	5.2	80%

It is important to note that column two contains the frequency range in which the reflexion coefficients were integrated and not the −10 dB frequency range.

## Methods

### Simulation

Numerical designs and simulations were performed using the commercial software CST Design Studio Suite. Periodic boundary conditions were applied in the numerical model in order to mimic a 2D infinite structure. Floquet ports were used for the excitation of the periodic structure. Simulation results were plotted using a free mathematical programming language tool. The calculation of the theoretical thickness using ${t}_{T}\ge |{\int }_{0}^{\infty }\,ln|\rho (\lambda )|d\lambda |/2{\pi }^{2}$ was done by mapping the simulated reflexion coefficients in the wavelength domain. The rectangle technique was used to calculate the integral in the formula.

### Measurements

**Figure 7:**
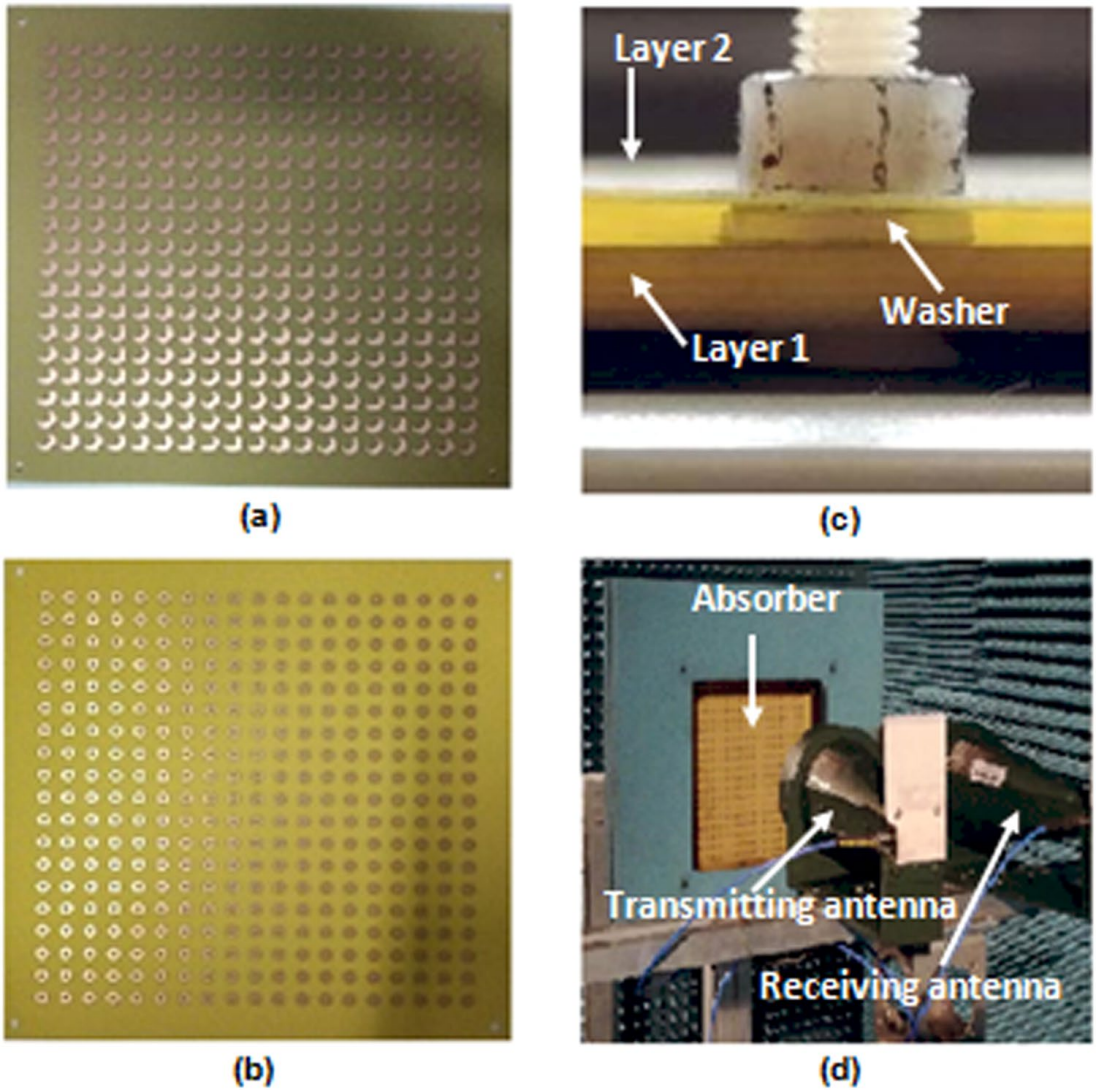
(**a**) HMSR layer. (**b**) RSR layer. (**c**) Fixing layers 1 and 2 with the 1.5 mm gap. (**d**) Measurement arrangements in anechoic chamber.

Measurements have been carried out in an anechoic chamber (Fig. 7d) using a vector network analyzer. A pair of broadband FLANNR horn antennas working in 2 GHz–18 GHz band was used as an emitter and receiver in reflection configuration. The reflection coefficient is normalized using the backed metallic plate of the structure acting as an ideal reflector. The sample is placed at a distance of 1 m in front of the antennas to satisfy far-field requirements. Experimental results were plotted using a free mathematical programming language tool.

### Data availability

The datasets generated and/or analyzed during the current study are not publicly available due to ongoing further studies and due to the industrial application of the project which is tested in an industrial setting. The corresponding information could be available upon reasonable request.

## Conclusion

An ultra-broadband thin FSS based radar absorber has been proposed achieving at least 89.7% of absorption in the whole band of 4.8 GHz–11.1 GHz. The absorption remains very interesting for oblique incidences. The absorption mechanism of our structure was first described by studying the contribution of each layer and secondly by observing the power loss density, the induced electrical and magnetic fields. Next, a parametric study complimented our work to show the coupling between the two layers and the effects that the air gap thickness has on the absorption. Another parametric study was done to give some optimized thicknesses of the two substrates and the air gap. The study also includes the presentation of a new approach to evaluate the performance of an absorber which consists of doing a ratio of the structure’s real thickness (calculated by its reflexion coefficient and frequency band) to its real thickness. After implementing measurements and calculations, our structure was found to have the best ratio when compared to some recent works on broadband absorbers. Eventually after fabricating the structure and analyzing it, the practical results proved to be in alignment with the numerical results. The experimental results are very promising and our design can be used to tackle EMI for civil and military applications.
